# Supplementary figures and images for: The limits of learning engagement and academic leadership within the higher education digitalization process ‐ analysis by using PLS SEM
Source: PLoS One. 2024 Nov 5;19(11):e0306079. doi: 10.1371/journal.pone.0306079 (PMC11537375; doi:10.1371/journal.pone.0306079)

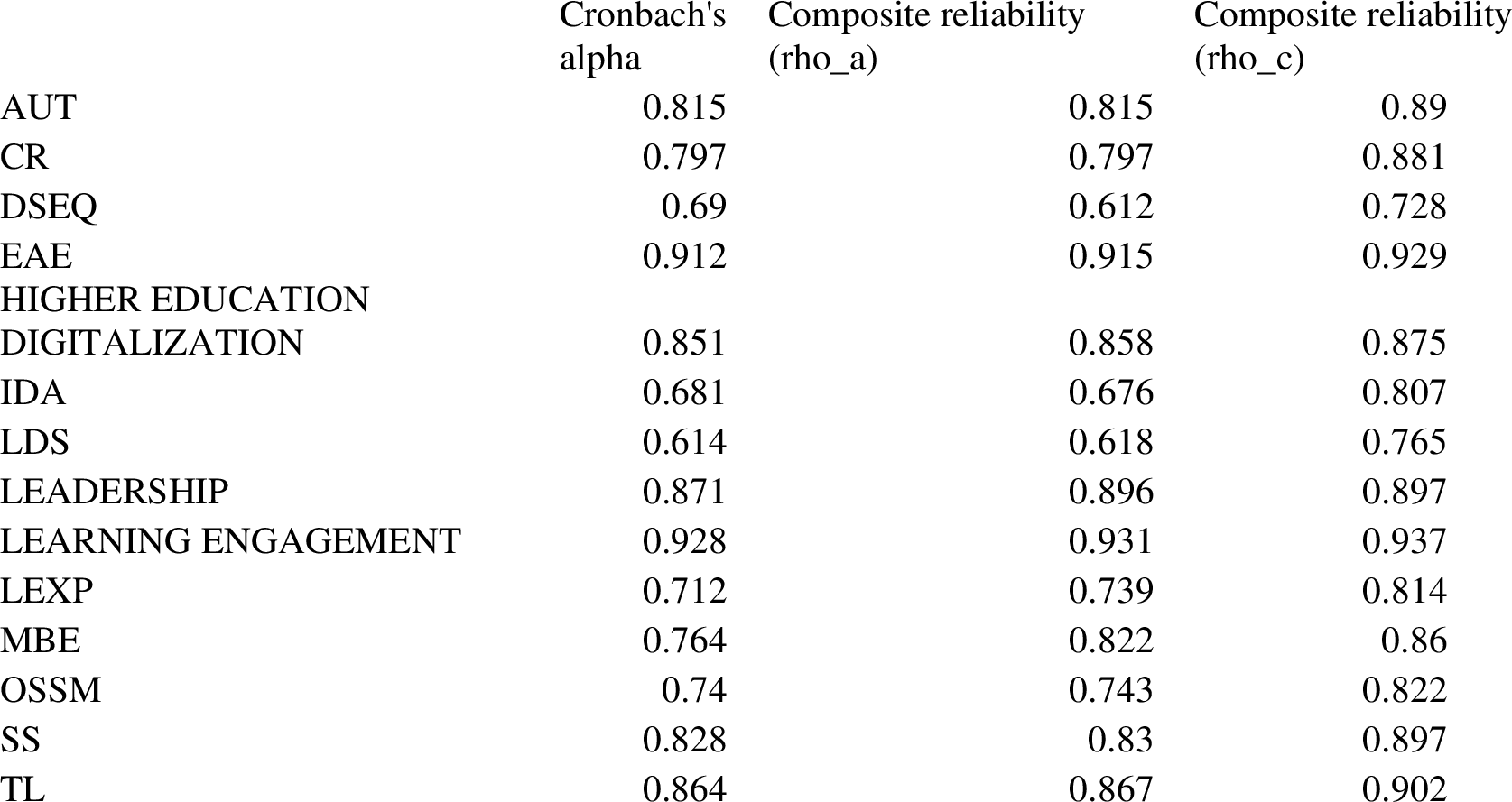

Supplement: S1 Table — Final values for the construct reliability and validity. (TIF) [file pone.0306079.s001.tif]

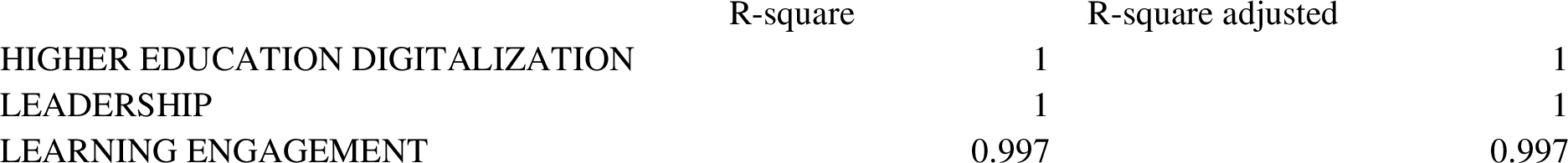

Supplement: S2 Table — Construct R^2 values. (TIF) [file pone.0306079.s002.tif]

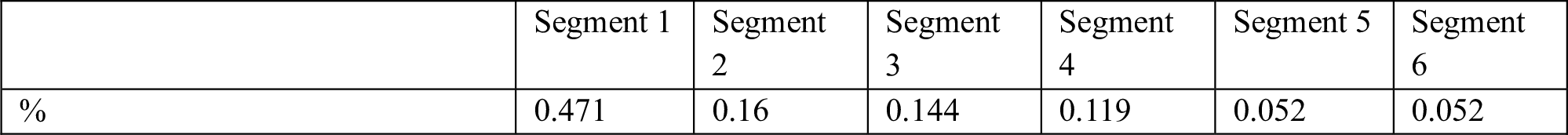

Supplement: S3 Table — Reference to the explanatory values of the six considered segment sizes. (TIF) [file pone.0306079.s003.tif]

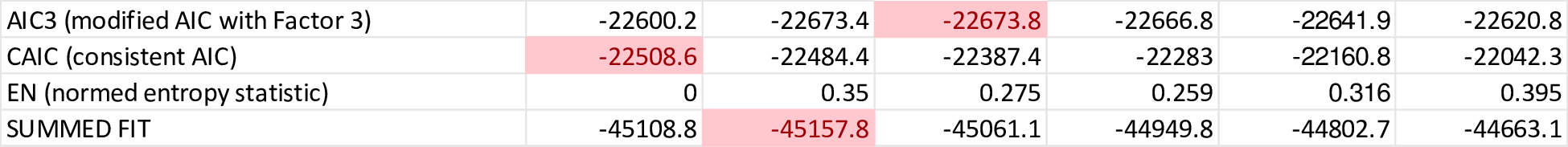

Supplement: S4 Table — Explaining the considered values for the FIMIX results. (TIF) [file pone.0306079.s004.tif]

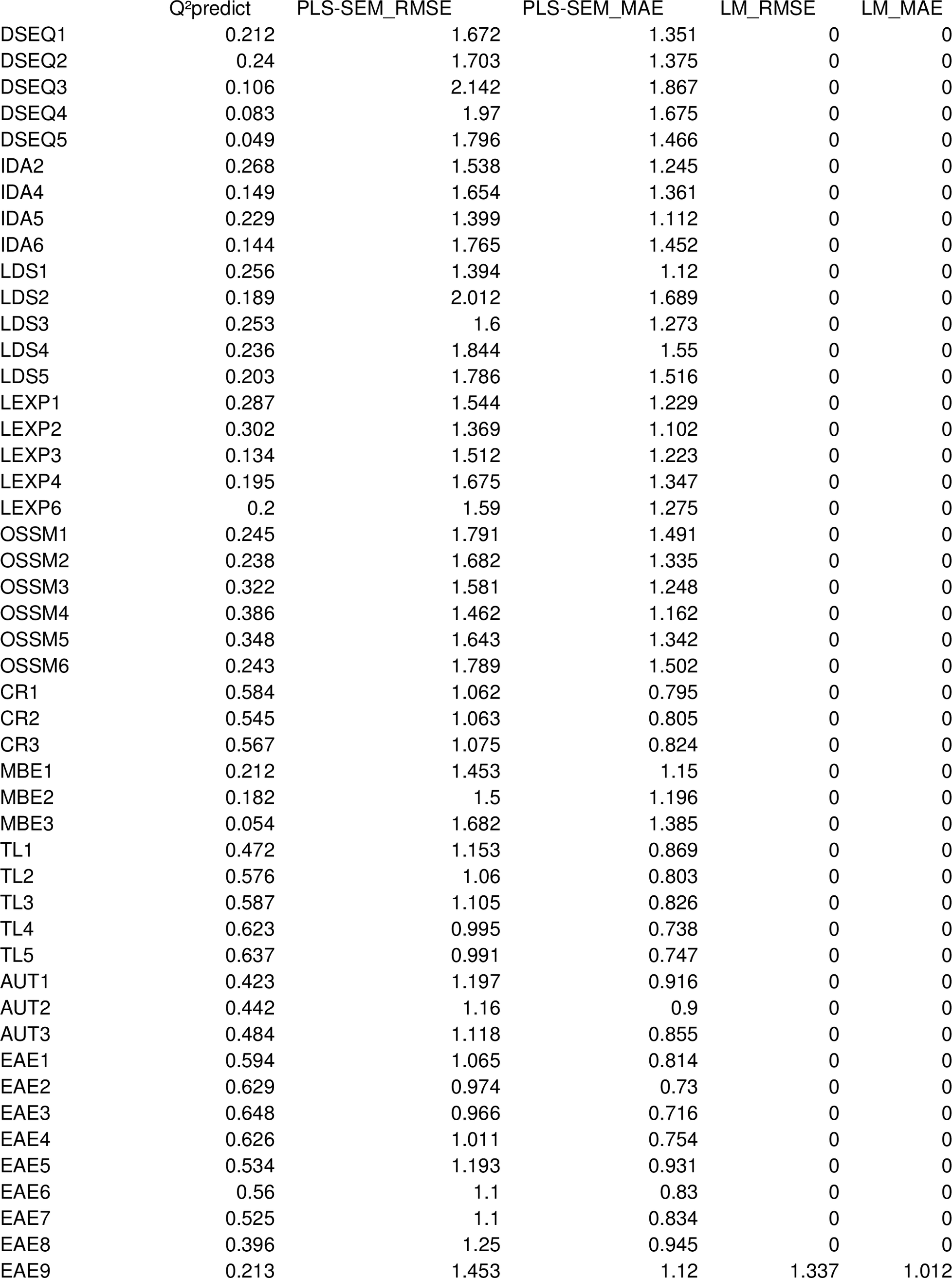

Supplement: S5 Table — Explaining the values for the PLS Predict analysis. (TIF) [file pone.0306079.s005.tif]

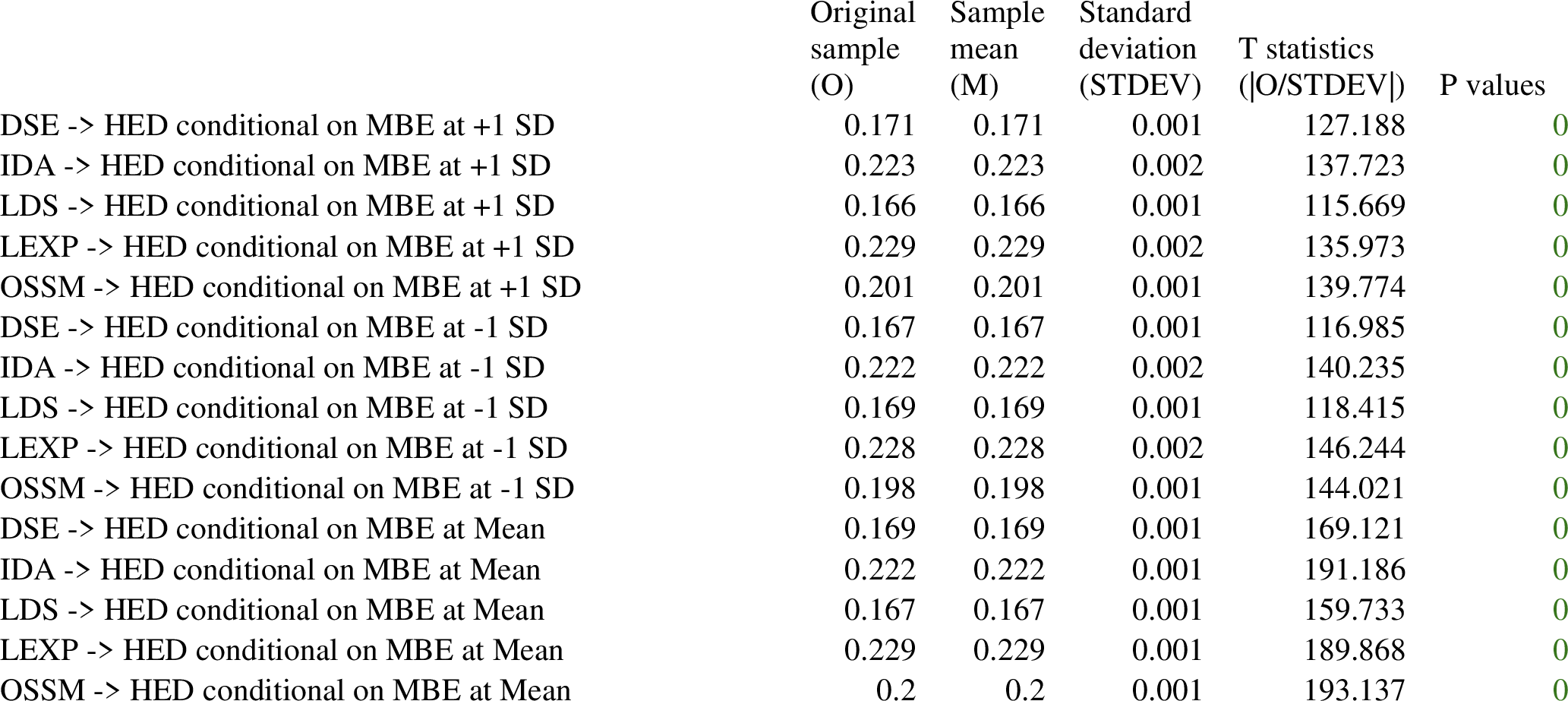

Supplement: S6 Table — Explaining the conditional direct effects. (TIF) [file pone.0306079.s006.tif]

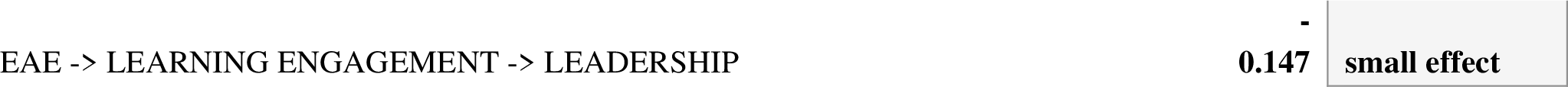

Supplement: S7 Table — Explaining the specific indirect effects. (TIF) [file pone.0306079.s007.tif]

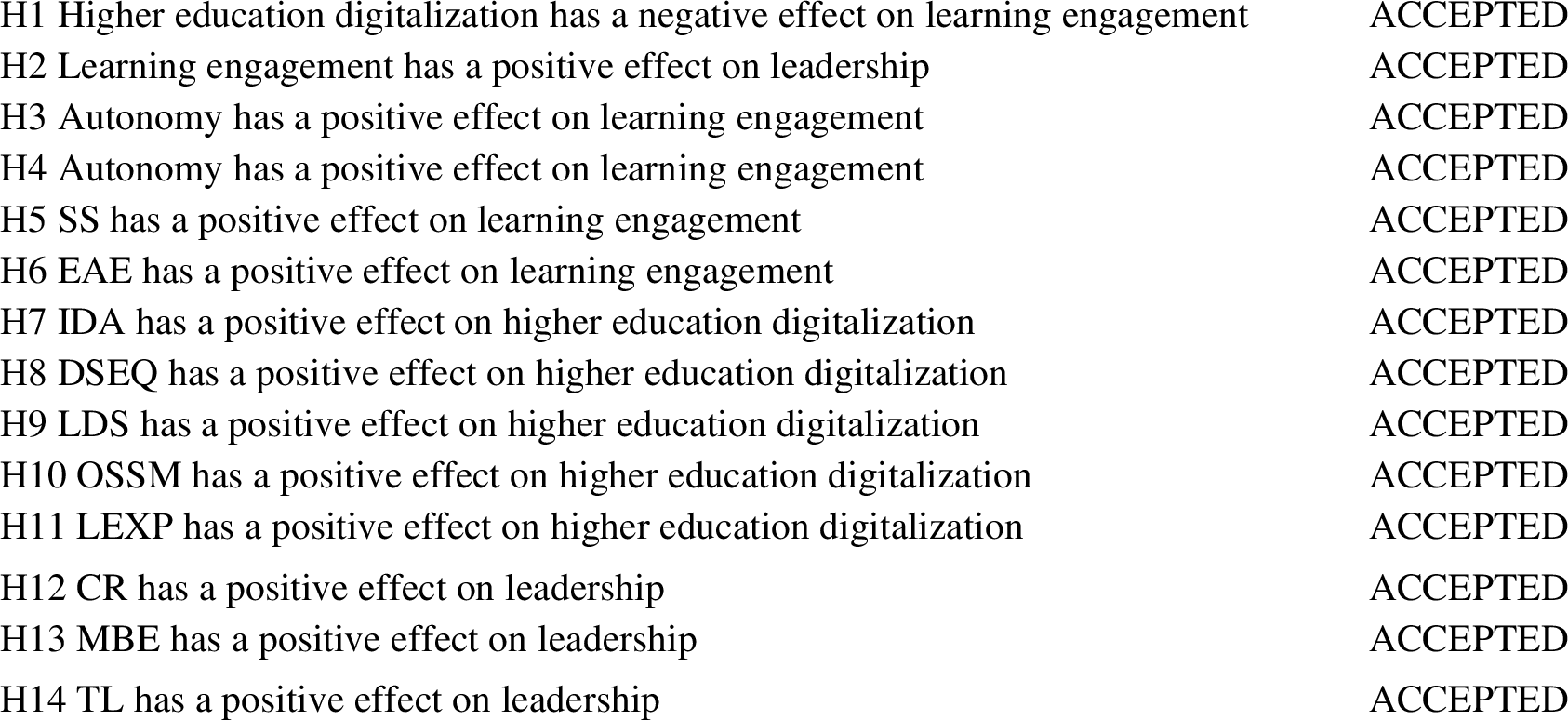

Supplement: S8 Table — Explaining the first results after testing structural hypotheses. (TIF) [file pone.0306079.s008.tif]

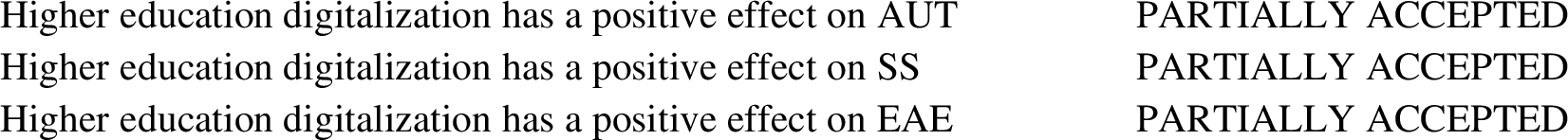

Supplement: S9 Table — Explaining the results for testing structural hypotheses (part 2). (TIF) [file pone.0306079.s009.tif]
