# Supplementary material for: The limits of learning engagement and academic leadership within the higher education digitalization process ‐ analysis by using PLS SEM
Source: PLoS One. 2024 Nov 5;19(11):e0306079. doi: 10.1371/journal.pone.0306079 (PMC11537375; doi:10.1371/journal.pone.0306079)
Supplement: Attachment — Submitted filename: Response to Reviewers.docx [file pone.0306079.s011.docx]

Reviewer #1:

Dear Reviewer,

Thank you for your kind support in regard to publishing our Manuscript.

As a response to your valuable insights, we introduced engagement and it’s types early within the foreword. Thank you for your suggestion.

As for the informed consent, the entire process and Ethical agreement approvals were already provided within the early submission stages. We included the necessary information within the Methodology section. Thank you for your suggestion.

Thank you very much for your support and valuable insights as for improving the quality of our manuscript.

Best regards,

Reviewer #2

Dear Reviewer,

We provided the suggested change within the Abstract. Thank you for your suggestion.

We replaced Foreword with Introduction.

We removed the duplicate of H1.

We explained with literature support the possible reasons for the high R^2 values.

We provided literature support for the small effect EAE -> LEARNING ENGAGEMENT -> LEADERSHIP, and discussed possible implications.

We adjusted the references as per Journal style.

We revised the entire methodology, including tables and figures.

Thank you very much for your support and valuable insights as for improving the quality of our manuscript.

Best regards,
